# Large Farm Animals Used for Research Purposes: A Survey on Purchase, Housing and Hygiene Management

**DOI:** 10.3390/ani11082158

**Published:** 2021-07-21

**Authors:** Tanja Schmidt, Fabienne Ferrara, Anne-Marie Pobloth, Sarah Jeuthe

**Affiliations:** 1 Institute for Animal Welfare, Animal Behavior and Laboratory Animal Science, Freie Universität Berlin, 14163 Berlin, Germany; 2 Consulting and Training in Laboratory Animal Science, 10589 Berlin, Germany; ff@consciencetrain.com; 3 Julius Wolff Institute, Charité-Universitätsmedizin Berlin, Corporate Member of Freie Universität Berlin, Humboldt-Universität zu Berlin, and Berlin Institute of Health, 13353 Berlin, Germany; annemarie.pobloth@googmail.com; 4 Max-Delbrück Centrum Berlin, 13125 Berlin, Germany; sarah.jeuthe@mdc-berlin.de

**Keywords:** farm animals, survey, preclinical research, agricultural research, hygiene monitoring, health

## Abstract

**Simple Summary:**

The use of farm animals, especially in biomedical research, has increased in recent years. As clear recommendations for the purchase, housing and health monitoring of these animals (sheep, goat, cattle and pigs) are still missing, many institutes have developed their own strategies and protocols to face the challenges associated with the use of farm animals. This may influence the comparability of research results and increase data variances, thus increasing animal use that contradicts the obligation to apply the 3Rs principle required in Directive 2010/63 EU and our national animal welfare law. Therefore, this survey aimed to define the current state of the art in research institutes working with farm animals in order to develop recommendations for the purchase, housing and hygiene management of farm animals used for research purposes; to refine the work with farm animals; and to reduce variability and, therefore, the number of animals required.

**Abstract:**

Background: Farm animals (FAs) are frequently used in biomedical research. Recommendations for the purchase, housing and health monitoring of these animals (sheep, goats, cattle and pigs) are still missing, and many institutes have developed their own strategies and protocols to face the challenges associated with the use of farm animals. This may influence the comparability of research results and increase data variances, thus increasing animal use that contradicts the obligation to apply the 3Rs principle of reduction, refinement and replacement required in Directive 2010/63 EU and the German animal protection law. Methods: A survey was conducted to define the current state of the art in research institutes working with pigs, and large and small ruminants. Results: The results of the survey clearly show that there are no uniform procedures regarding the purchase, housing and hygiene management of farm animals contrary to small laboratory animals. The facilities make purpose-bound decisions according to their own needs and individual work instructions and implement their own useful protocols to improve and maintain the health of the animals. Conclusion: This survey was the first step to filling the gaps and identifying the status quo and practical applied measures regarding the purchase and hygiene monitoring of FAs in order to improve animal welfare and scientific validity.

## 1. Introduction

Farm animals (FAs) are widely used in scientific research for studies related to animal husbandry, agricultural and veterinary science, basic safety and comparable and translational medicine [1,2,3]. The anatomical structures, size and function of many organs are largely comparable between FAs and humans, qualifying FAs as highly interesting models for the study of human diseases, e.g., the use of pigs in cardiovascular or transplantation research [4,5] or the use of sheep in the field of reproductive science and as a surgical model in orthopedic studies [6,7,8]. Furthermore, transgenic FA models are increasingly available and are becoming frequently used [9,10]. FAs are also useful comparative models; they offer the opportunity to generate results with high biological relevance and, in various instances, are even of dual use in the sense that they foster the improvement of human as well as animal health [11].

According to European legislation, small laboratory animals must be bred specifically for use in animal experiments and, therefore, obtained from commercial laboratory animal breeders [12].

In contrast to small laboratory animals, FAs are usually obtained directly from livestock production [13], resulting in remarkable heterogeneity with respect to genetic background, microbiological load and hygienic status and, consequently, animal health [4]. Comparable and widely used standardized guidelines for health and hygienic standards have been lacking in recent years [13,14]. These factors may adversely affect animal welfare and, therefore, the scientific outcome. his is in contrast to the legal requirements in accordance with Annex III (requirements for establishments and for the care and accommodation of animals) of Directive 2010/63/EU on the protection of animals used for scientific purposes, where it is required that animal facilities have a strategy in place to ensure that the health status of the animals is maintained, and this strategy includes regular health monitoring and a microbiological surveillance program (Annex III, Section A, paragraph 3.1.) [12]. As an example, zoonotic pathogens, such as Coxielle burnetii, the causative agent of Q-fever [15] or infections with poxviruses in small ruminants, are common in livestock production. If these pathogens are introduced in an animal facility, they can seriously affect human and animal health [4,16]. While notifiable and reportable animal diseases are usually controlled by national authorities (in accordance with EU regulations and recommendations given by the World Health Organization, WHO, and the World Organisation for Animal Health, OIE) many potential pathogens are less controlled or even neglected on the farm side. In terms of research, unknown or clinically latent infections with pathogens may adversely affect animal welfare and, therefore, influence the scientific outcome, as data variability and the drop-out rate may increase [16]. One example is the ubiquitous but widely underdiagnosed presence of *Chlamydia* species with a remarkably broad host range, including pigs, cattle and small ruminants, and a high proportion of latent infections that do not lead to overt disease [17,18]. Nevertheless, persistent and/or recurrent chlamydia infections in pigs, calves and adult cattle were proven to be significantly associated with chronic effects and animal health at a subclinical level [17,19,20,21]. 

In 2015, the LaNiV (Landwirtschaftliche Nutztiere in der Versuchstierkunde) network was funded by farm animal experts in veterinary medicine, agricultural research and biomedicine science working in Germany, Switzerland and Austria in order to engage in the exchange of knowledge in the sense of the 3Rs principle [22]. One target of the working group was to define recommendations to control health and hygienic status in FAs housed under experimental conditions and used for research. The first step was to identify the current methods of purchase, housing and health and hygienic monitoring in experimental housing in Germany. Therefore, we conducted a survey to establish the basis to develop recommendations for the future.

## 2. Materials and Methods

### General Remarks

The survey was divided into two main sections, a general part and a species-specific part. The general questions (section A) addressed nine topics on the research focus, housed species (pigs, small ruminants and large ruminants), purchase, general housing conditions, hygiene management and procedures at the end of the experiments.

In section B, the species-specific part, we focused on information on the hygiene-monitoring protocols in the participating facilities. For this purpose, we provided our participants with a detailed species-specific list with all reportable and notifiable animal diseases and pathogens in Germany and with all pathogens from the Federation of the European Laboratory Animal Science Associations’ (FELASA) recommendations for the health monitoring of pigs, calves, sheep and goats from 1998 [16] to 2000 [23]. 

All answers had to be provided individually for each animal species (pig, small ruminant and large ruminant) if present. The survey was provided in the German language on the surveymonkey.com platform and was available from 2 February to 10 March 2017. It was distributed with the aid of the German animal welfare officer mailing list, the LANIV network and the German wide animal house manager mailing list. Furthermore, it was announced on the webpage of the animal welfare officers as well as on the LANIV network webpage. The survey was anonymous and the participants could add comments while answering some of the questions as well as at the end of the survey. After collecting all answers, data were exported to an Excel workbook. Each survey was individually screened for the percentage (%) of answered questions by one of the authors. Surveys with more than 44% answered questions in section A were included for further evaluation. For detailed information on the general part of our survey, all questions and answer possibilities are provided in the Supplementary Materials (Table S1). 

## 3. Results

Due to the low participation rate in the species-specific part (section B), only the data of the general section (section A) are presented here. Of the 44 participants who took part in the survey, 14 surveys were excluded due to incomplete data sets with less than 40% answered questions. In total, 30 surveys were included and evaluated. On average, the included participants answered 81% of the questions. Open fields were classified as “no information”.

In the included surveys, pigs were the most frequently housed farm animal species (*n* = 28) followed by small ruminants (*n* = 23) and large ruminants (*n* = 9). All the following results are based on these datasets. 

**Question 1:**

Please provide detailed information about the nature of your facility, the housed species, the housing capacities and the age groups of the housed animals. 

**Pigs and small ruminants**

**Figure 1:**
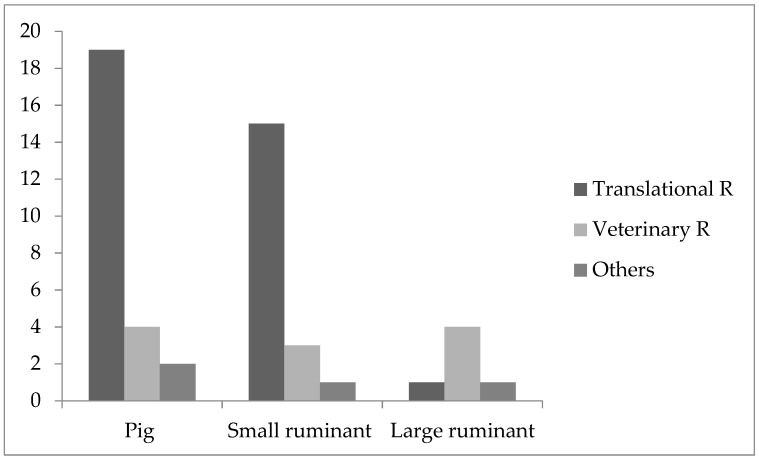
Medical research. The figure shows the specific use of farm animals in the field of biomedical research. The comments of participants indicated that “others” may include education and training purposes.

The majority of the participants used pigs and small ruminants in the field of medical research (pigs = 25; small ruminants = 19) with a focus on translational research (pigs = 19; small ruminants = 15), followed by veterinary research (pigs = 4; small ruminants = 3) and other fields (pigs= 2; small ruminants = 1; Figure 1). 

**Figure 2:**
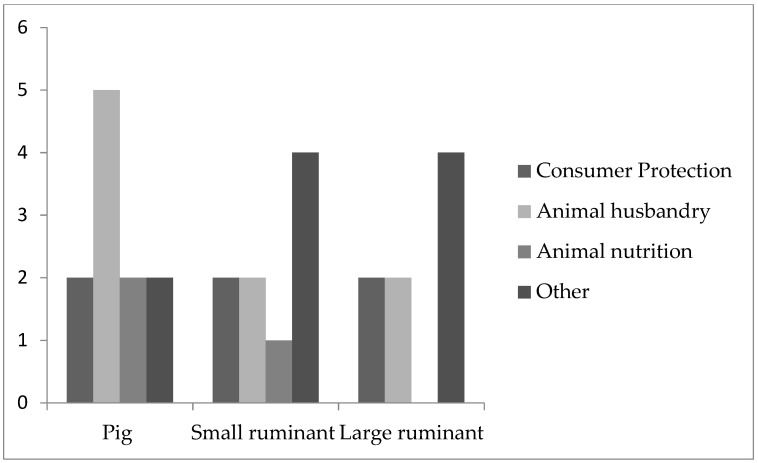
Agricultural research. The figure shows the specific use of farm animals in the field of agricultural research. Based on the comments of our participants, “others” may mainly include animal health, animal behavior, infection medicine and diagnostics of animal diseases.

Pigs used in agricultural research (*n* = 11; Figure 2) were mostly used for research addressing animal husbandry (*n* = 5). Two participants mentioned using pigs in consumer protection research, animal nutrition or other fields. 

Small ruminants used for agricultural research (*n* = 9) were mostly used in other fields (*n* = 4) followed by consumer protection (*n* = 2), animal husbandry (*n* = 2) and animal nutrition (*n* = 1) (Figure 2).

**Large ruminants**

Large ruminants were more often used in agricultural research (*n* = 8) compared to medical (*n* = 6) research. In medical research (Figure 1), most participants used large ruminants for veterinary research (*n* = 4) followed by translational research and others (each = 1). In agricultural research, large ruminants were mostly used in other fields (*n* = 4), followed by consumer protection (*n* = 2) and animal husbandry (*n* = 2) (Figure 2). 

Comments

In the general comments, fourteen participants mentioned using farm animals for teaching purposes, i.e., either in the translational research field for surgical courses or in animal experimentation courses for students, animal caretakers and researchers. In translational research, cardiology, orthopedics and regenerative medicine, vaccination development, plasma proteins and coagulation, imaging, anesthesia, biotechnology and testing of medical devices were reported. In agricultural or veterinary research, animal nutrition and health, housing, behavior, infection medicine and diagnostics of animal diseases were specified.

A total of 16 participant institutions were also breeding facilities, predominantly breeding for their own use, but also including two commercial breeders (pigs and small ruminants). 

Furthermore, we asked for the age groups and total housing capacities of animals (Table 1 and Table 2). If participants responded, they provided information on both topics; however, the total number of answers was low regarding these topics (Table 1 and Table 2). Most of the facilities housed young and adult animals. For small ruminants, some facilities kept only adult animals. This could not be observed in facilities that kept pigs and large ruminants.

**Question 2:**

What are the access restrictions in your livestock facilities? 

**Pigs**

Most participants had restricted access (yes = 25; no = 3) and changed their clothing and shoes (yes = 24; no = 4). Nine of the participants described their facility as a strike barrier (yes = 9; no = 17; no information = 2), three indicated working with showers (yes = 3; no = 23; no information = 2). Only seven participants reported having no access barriers (yes = 7; no = 19; no information = 2). 

**Small ruminants**

Most participants had restricted access (yes = 19; no = 3; no information = 1) and changed their clothing and shoes (yes = 17; no = 5; no information = 1). Two of the participants described their facility as a strike barrier (yes = 2; no = 19; no information = 2). No participant indicated working with showers (yes = 0; no = 22; no information = 1). Only five participants reported having no access barriers (yes = 5; no = 16; no information = 2). 

**Large ruminants**

Only one participant declared their facility as having restricted access (yes = 1; no = 8; no information = 0), but eight participants stated that they changed their clothing and shoes (yes = 8; no = 1; no information = 0). None of the participants described their facility as a strike barrier (yes = 0; no = 9; no information = 0) or indicated working with showers (yes = 0; no = 9; no information = 0). Two participants reported having no access barriers (yes = 2; no = 6; no information = 1). 

**Question 3:**

Please describe your methods for germ reduction, in particular your ventilation and the type of water and feed used, as well as if roughage and green feed is provided. 

**Pigs**

Six participants did not use any ventilation system in their facilities or did not provide information about the presence of a ventilation system. Eight participants reported having ventilation with overpressure and a HEPA-filtered air supply (*n* = 5) or without a HEPA-filtered air supply (*n* = 2). Eight participants reported using vacuum pressure ventilation with a HEPA-filtered air supply (*n* = 4) or without a HEPA-filtered air supply (*n* = 4). 

In most of the facilities, untreated tap water was provided to the animals for drinking (*n* = 22), four supplied germ-reduced tap water and two did not provide information. 

Most participants reported using standardized finished feed (*n* = 24) and four reported that they do not use finished food. 

Twelve participants stated that they fed their animals roughage, one stated that they fed their animals germ-reduced roughage, two did not feed their animals roughage and 13 did not specify what they fed their animals. Three of the participants stated that they fed their animals green feed, 11 did not feed their animals green feed and 14 did not provide specific information.

**Small ruminants**

Most participants reported that there was no ventilation system in their facilities (*n* = 8) or did not provide information (*n* = 9). In four facilities, a ventilation system was present, either with overpressure and a HEPA-filtered air supply (*n* = 3) or without a HEPA-filtered air supply (*n* = 1). Two participants reported using vacuum pressure ventilation with a HEPA-filtered air supply.

Most of the participants supplied untreated tap water to the animals (*n* = 16), two used germ-reduced tap water and five did not provide information. 

Most participants reported using standardized finished feed (*n* = 18), three reported that they did not use finished food and two did not provide any information. Ten of the participants stated that their animals received roughage, two did not feed their animals roughage and 11 did not provide specific information. Four of the participants stated that they fed their animals green feed, six did not feed their animals green feed and 13 did not provide specific information.

**Large ruminants**

Most participants did not provide information (*n* = 6). One participant declared the absence of a ventilation system in the facility (*n* = 1). Two participants reported having a ventilation system: One with overpressure and one with negative pressure, but both with a HEPA-filtered air supply.

In three facilities, untreated tap water was supplied to the animals (*n* = 3). One participant used germ-reduced tap water and five did not provide any information. Three participants reported using standardized finished feed (*n* = 3), but six did not provide any information. One participant stated that they fed their animals roughage, but eight did not provide specific information. One of the participants stated that they did not feed their animals green feed and the other eight did not provide specific information.

**Question 4:**

Please provide information regarding whether you purchase the animals from a special breeder for experimental use or from livestock production.

**Pigs**

Most of the participants purchased pigs from livestock production breeders (Table 3, *n* = 23; no information = 5), mostly consistently from one livestock farm (*n* = 14).

Three participants indicated that they purchased pigs from an animal trader. Two participants received pigs from various livestock farms. One participant consistently purchased pigs from both a livestock farm and a breeder of experimental animals. One participant stated that they purchased pigs from a livestock farm and also bred pigs themselves. One participant indicated that they obtained pigs from various livestock farms and also bred pigs themselves. Another participant purchased pigs from various livestock farms and a trader. 

Fifteen participants indicated that they obtained pigs from a special breeder (*n* = 15; no information = 13), mostly from one particular specialized breeder of experimental animals (*n* = 7), and also bred pigs themselves (*n* = 4). 

Two participants purchased their pigs from various breeders of laboratory animals. Two participants indicated that they bred their own pigs but also purchased animals from various experimental animal breeders or animal traders.

Due to the possibility of leaving a comment in answer to this question, we received one answer stating that the origin of pigs depends on the number of animals that need to be ordered and their availability.

**Small ruminants**

From the participants who obtained small ruminants from a special breeder (Table 4, *n* = 11; no information = 12), most participants also bred animals themselves (*n* = 5). Two participants stated that they bred their own animals and purchased animals from one breeder of experimental animals as well. Two participants purchased small ruminants from one breeder of experimental animals. Two participants purchased small ruminants from various specialized breeders of animals used for experimental purposes. 

From the participants that purchased small ruminants from livestock production breeders (*n* = 18; no information = 5), the majority (*n* = 11) indicated that they obtained animals consistently from one farm. Two participants responded that they purchased small ruminants from various farms and two participants obtained small ruminants from a trader who obtained animals from various livestock farms. One participant indicated that they bred their own animals and purchased them from various livestock farms. Another participant bred their own animals and purchased animals from a trader who obtained small ruminants from a livestock farm.

**Large ruminants**

From the participants who stated that they obtained large ruminants from special breeders (*n* = 6; no information = 3), most participants served as their own breeding facility (*n* = 5). One participant bred animals independently and purchased animals used for experimentation from a specialized breeder. 

From the participants who purchased large ruminants from livestock production (*n* = 7; no information = 2), three participants indicated that they bred their own large ruminants and also purchased large ruminants from one farm. Two participants consistently purchased large ruminants from one farm. One participant stated that they purchased large ruminants from various farms. One participant received animals from a trader who obtained animals from various unknown livestock farms. 

**Question 5:**

Please describe the health status and hygiene monitoring performed when purchasing animals.

1. Are the animals transported separately when obtained from different origins?

**Pigs and small ruminants**

When the animals were purchased from different origins, transportation of pigs and small ruminants was mostly performed separately (pigs: Transport separate = 22; transport not separate = 1; no information = 5; small ruminants: Transport separate = 16; transport not separate = 2; no information = 5).

**Large ruminants**

Large ruminants were more frequently transported together when purchased from different origins (transport separate = 2; transport not separate = 3; no information = 4).

2. In general, do you purchase animals with or without a health certificate? Furthermore, please describe if the certificate includes information about disease history and serological, parasitological or bacteriological results.

**Pigs**

**Figure 3:**
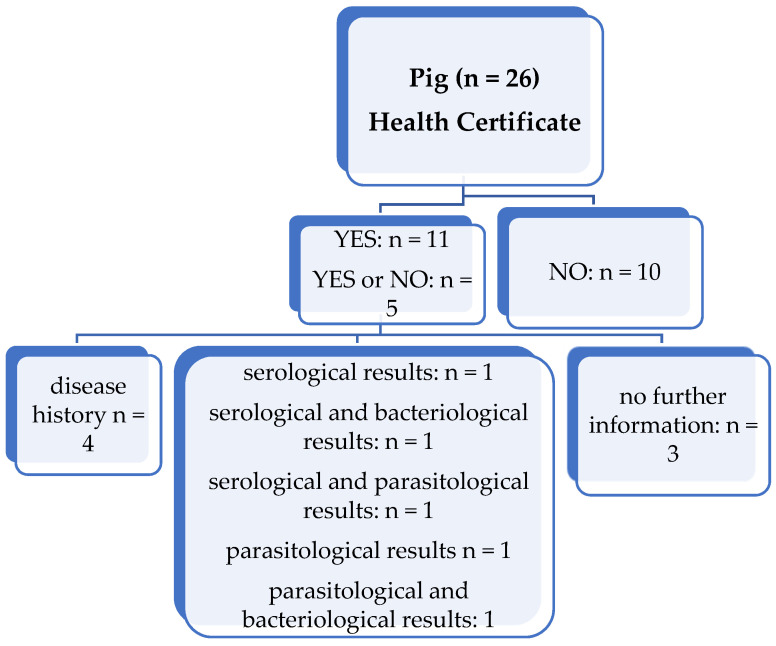
Overview of whether pigs were purchased with or without a health certificate and which information was provided in the health certificate. Two participants did not answer the question. *n* = number of responding participants; yes/no: Testing was/was not performed.

Ten participants purchased pigs without health certificates, eleven participants purchased pigs with health certificates and five participants answered that they purchased pigs both with and without health certificates (Figure 3). Two participants provided no information.

Regarding the participants who purchased pigs with health certificates, most participants (*n* = 4) received a health certificate that included only the disease history and no further information on the serological, parasitological or bacteriological results. Three participants received the serological results, and one of them also included either the bacteriological or parasitological results. One participant received parasitological results and another parasitological and bacteriological results. Three participants gave no further information on the included analysis. 

In the comments, it was noted that miniature pigs were specifically obtained in a pathogen-free state, according to the FELASA recommendations. 

**Small ruminants**

**Figure 4:**
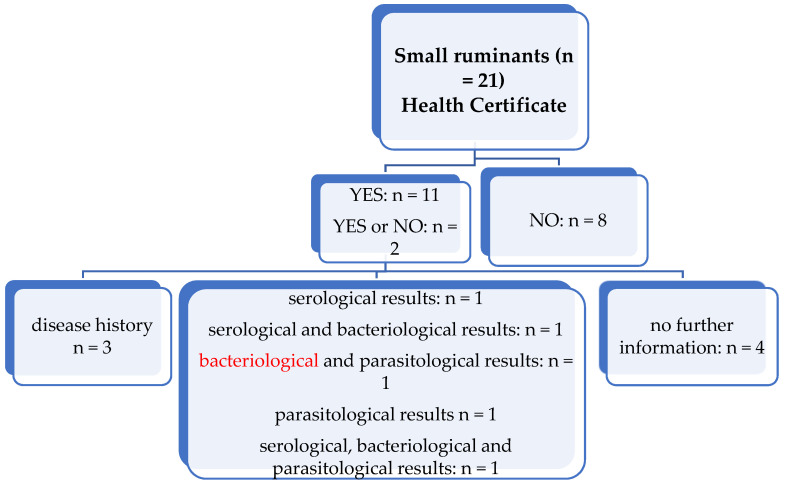
Overview of whether small ruminants were purchased with or without a health certificate and which information was provided in the health certificate. Two participants did not answer the question. *n* = number of responding participants; yes/no: Testing was/was not performed.

Eight participants generally purchased small ruminants without a health certificate, eleven participants purchased small ruminants with a health certificate and two participants answered that they purchased small ruminants both with and without a health certificate (Figure 4). Two participants provided no information.

Regarding the participants who received a health certificate, most participants (*n* = 4) received a health certificate without the disease history, serological, parasitological or bacteriological results. Three participants received a health certificate that included only the disease history. Two participants received a health certificate with serological results and one of them received a certificate with additional bacteriological results. Two participants received a health certificate with parasitological results and one of them received a certificate with additional bacteriological results. Only one participant received a health certificate with serological, parasitological and bacteriological results. One participant gave no further information on the analyses included in the certificate.

It was commented that rams are purchased with a health certificate stating their current status. 

**Large ruminants**

Four participants generally purchased large ruminants without a health certificate and two participants purchased large ruminants with a health certificate. Three participants provided no information.

Regarding the participants who received a health certificate, one participant received a health certificate with the disease history and the other participants received a health certificate without any further information.

It was commented that when stock bulls are purchased, a health certificate with their current status is mandatory.

Comments

A few participants commented that the farm from which they consistently purchase animals monitored the livestock to determine their health status. In small ruminants and pigs an endoparasite treatment, and in small ruminants an ectoparasite treatment, are performed routinely before transportation in some cases.

3. Does your health certificate include any information about the exclusion of a pregnancy and about their/other measures to avoid/exclude an unwanted pregnancy?

**Pigs**

Regarding the 16 participants who received a health certificate, most participants (*n* = 9) received a health certificate without the exclusion of pregnancy. Four participants received a health certificate with the exclusion of pregnancy and three provided no information. 

Regarding all participants who housed pigs, most participants (*n* = 14) stated that they housed different sexes separate from each other to prevent unwanted pregnancies without performing additional ultrasound examinations or medicinal treatments. Eight participants answered that they performed no ultrasounds, medicinal treatments or separate housing of different sexes after delivery to detect or avoid a pregnancy. Six participants provided no information. 

**Small ruminants**

Regarding the 13 participants who received a health certificate, most participants (*n* = 9) answered that the health certificate did not exclude pregnancy. Three participants received health certificates with the exclusion of pregnancy and one provided no information. 

Regarding all participants housing small ruminants, most participants (*n* = 7) responded that they housed different sexes separate from each other to prevent unwanted pregnancies and without performing additional ultrasound examinations or medicinal treatments. Five participants answered that they performed no ultrasounds, medicinal treatments or separate housing of different sexes after delivery to detect or avoid pregnancies. Three participants responded that they performed ultrasounds and medicinal treatments, and did house different sexes separately. Two participants stated that they performed ultrasounds and had separate housing for different sexes to avoid pregnancies. Five participants provided no information on any aspect of the housing of small ruminants. 

**Large ruminants**

Regarding the two participants who received a health certificate, one of them responded that they received a certificate that included the exclusion of pregnancy, while the other participant received a certificate without this information. 

Regarding all participants housing large ruminants (*n* = 9), most participants (*n* = 3) responded that they performed no ultrasound or medicinal treatments and did not house different sexes separately. One participant responded that they did house different sexes separately. Five participants provided no information on any aspect of the housing of large ruminants. 

Comments

As a comment, we received the answer that (in contrast to the exclusion of pregnancy), when pregnant farm animals are ordered, a certificate of pregnancy is mandatory. 

**Question 6:**

The quarantine procedure, prophylactic treatment management and the restocking of the housing rooms were queried.

1. Quarantine procedures

**Pigs**

While 13 facilities did not report the use of any quarantine program, 15 facilities confirmed that they quarantined the animals. Pigs were mostly quarantined routinely in their own holdings after delivery (*n* = 8), while quarantining was also performed in the holdings of origin either in suspected cases (*n* = 4) or routinely (*n* = 3) according to other participants. 

**Small ruminants**

In 12 facilities, animals were quarantined in their own holdings either routinely (*n* = 8) or in suspected cases (*n* = 4). Nine participants answered that no quarantine procedures were carried out and two users provided no information. 

**Large ruminants**

In five facilities, large ruminants were quarantined in their own holdings either routinely (*n* = 2) or only in suspected cases (*n* = 3). Two participants answered that they had no quarantine procedures or provided no information.

2. Prophylactic treatments and restocking procedures of housing rooms (in–out principle or no in–out principle)

**Pigs**

Most participants (*n* = 18) performed prophylactic treatments; however, regarding choice, frequency and location, no clear trend was visible. Antiparasitic treatments alone were performed in five facilities (in participants’ own holdings—single treatment (*n* = 1) or repeated (*n* = 2); or in the holdings of origin—single treatment (*n* = 2)). Vaccinations alone were performed in four facilities (in participants’ own holdings—repeated (*n* = 1); or in the housing of origin—single (*n* = 1) or repeated (*n* = 2). 

Combined treatments were confirmed by nine participants (in participants’ own holdings—single treatment (*n* = 1) or repeated treatments (*n* = 3); or in the holdings of origin—single (*n* = 3) or repeated (*n* = 2)). 

No prophylactic treatments were confirmed by eight facilities. Two participants provided no information. 

In most of the facilities (*n* = 22), new occupancy of the housing rooms was approached following the in–out principle, meaning that after occupation by one animal group, the room was disinfected before new animals were introduced. Three facilities did not restock following the in–out principle and three did not provide any information. 

**Small ruminants**

Nineteen participants confirmed that prophylactic treatments were applied, while their frequency and application varied. Prophylactic antiparasitic treatments alone were applied in 12 facilities (in participants’ own holdings—single treatment (*n* = 2) or repeated treatments (*n* = 4); or in the holdings of origin—single treatment (*n* = 5) or repeated treatments = 1)) compared to vaccinations alone, which were applied in two facilities (in participants’ own holdings—repeated treatments). Five participants combined antiparasitic treatments and vaccination (in the holdings of origin—single (*n* = 1) or repeated treatments (*n* = 1); or in participants’ own holdings—repeated treatment = 3). 

Two respondents answered that they did not perform any treatments and two participants did not provide any information. 

The restocking of housing rooms for small ruminants followed the in–out principle in 13 facilities. In eight facilities, the in–out principle was not applied and two users did not provide any information on restocking procedures.

**Large ruminants**

Prophylactic treatments were carried out in five facilities in participants’ own holdings after the entry of the animals to the research premises. Single (*n* = 2) prophylaxis or repeated antiparasitic treatments (*n* = 1) were mainly performed. In only one facility were vaccinations or combinations of vaccinations and antiparasitic treatments performed. Four participants provided no information on prophylactic treatments. No trend regarding restocking procedures was visible (yes: 3; no: 4; no information: 2). 

**Question 7:**

Are health-monitoring programs for farm animals established in your facility? 

Do you perform a necropsy after the death of an animal to monitor health issues and, if so, is this carried out internally or externally? 

**Pigs**

Most of the participants (*n* = 26) confirmed that they had an established health-monitoring program that is performed routinely in 17 facilities, while nine facilities only followed a health-monitoring program in suspected cases. Only one facility had no health-monitoring program or provided no information. 

In most of the facilities, autopsies in pigs were performed in house in suspected cases (*n* = 14). In six facilities, in-house necropsies were performed routinely. Five participants mentioned that they did not perform in-house necropsies and three provided no information. 

An examination of the sacrificed pigs in external institutes was performed only in suspected cases in 13 facilities, whereas nine facilities never performed external necropsies. Six participants provided no information.

**Small ruminants**

Most of the participants (*n* = 20) confirmed the presence of an established health-monitoring program in their facilities, which was either performed routinely (*n* = 12) or in suspected cases only (*n* = 8). Three facilities had no health-monitoring program.

In-house necropsies were performed in most of the facilities in suspected cases (*n* = 12), while some routinely performed necropsies (*n* = 4). In six facilities, no in-house pathology was performed. One participant provided no information.

Autopsies of small ruminants in external institutes was reported, but only in suspected cases (*n* = 15). Five participants answered that their facilities never demanded external necropsies. Three participants did not provide any information. 

**Large ruminants**

Eight participants confirmed that they carried out routine monitoring of animal health within their facility. In one facility, health examinations were only performed in suspected cases. 

Necropsies were performed externally more often (*n* = 7), versus in-house (*n* = 3) autopsies that were performed in suspected cases only. Examinations of animals’ external pathology were reported on two occasions, in suspected cases (*n* = 3) and routinely (*n* = 4). Five participants mentioned that they never performed necropsies of large ruminants in house. Two participants mentioned that they never required external pathological examinations of large ruminants. One participant provided no information.

**Question 8:**

What type of livestock animal husbandry is used? 

**Pigs**

Most participants housed their pigs in groups in a stable all year round with (*n* = 14) or without (*n* = 8) the possibility of single housing. One participant stated that pigs were housed in single boxes in the stable all year round (*n* = 1). Two participants reported the possibility of group housing in the stable with year-round pasture keeping and the possibility of separate housing of individual animals (*n* = 2). Three participants did not provide information. 

**Small ruminants**

The majority of the participants reported that they housed small ruminants in groups in a stable with temporary pasture keeping (*n* = 7) or in groups in a stable all year round with the possibility of single housing (*n* = 4). Two participants reported that they housed small ruminants in groups in a stable (*n* = 2) or kept them in a pasture all year round with (*n* = 2) or without the possibility of single housing (*n* = 2). Six participants did not provide information. 

**Large ruminants**

From the nine participants that answered the question, one reported housing their large ruminants in groups in a stable with temporary pasture keeping (*n* = 1). Eight participants did not provide information.

**Question 9:**

Have you established any kind of internal or external program to share organs and tissues? 

Are animals released, rehomed or returned to the food chain after the end of procedures?

1. Sharing Organs and tissue

**Pigs**

The majority (*n* = 22) of the participants reported that they use programs for sharing the organs and tissues of sacrificed animals. This result was crosslinked to the only use of institutional (internal) sharing programs. Two participants provided no information and four participants used neither an internal nor an external program.

**Small ruminants**

A total of 15 participants confirmed the use of programs to share organs and tissues. As with pigs, only internal programs were used. Only one user provided no information and seven participants used neither an internal nor an external program. 

**Large ruminants**

In total, five participants endorsed the use of sharing programs. In three cases, this was related to internal programs and two participants confirmed the use of external programs. Two participants used neither an internal nor an external program and one participant did not provide information.

2. Animal release, rehoming or return to food chain

**Pigs**

The majority of participants did not give animals away after the end of use (*n* = 17). Private release (rehoming) was confirmed in four cases and commercial release to return pigs to the food chain (external slaughtering) was confirmed in five cases. However, only three participants confirmed that they carried out commercial animal release and external slaughtering with the presence of an official veterinary certification. In addition, a total number of four participants confirmed that they carried out internal slaughtering. Two participants did not provide any information.

**Small ruminants**

Most of the participating facilities did not give animals away after the end of their use, neither privately nor commercially (*n* = 15). Private release (rehoming) was confirmed in four cases and commercial release to return small ruminants to the food chain (external slaughtering) was confirmed in four cases. However, only two participants confirmed that they carried out commercial animal release and external slaughtering with the presence of an official veterinary certification. In addition, a total number of four participants confirmed that they carried out internal slaughtering. One participant did not provide information.

**Large ruminants**

In total, two participants stated that they do not give animals away after the end of their use, neither privately nor commercially. Private release (rehoming) was confirmed in one case and commercial release to return large ruminants to the food chain (external slaughtering) was confirmed in five cases. Three participants confirmed that they carried out commercial animal release and external slaughtering with the presence of an official veterinary certification. In addition, a total number of two participants confirmed that they carried out internal slaughtering. Two participants did not provide information.

## 4. Discussion

The aim of this survey was to gain detailed information on the handling and management of using FAs in research facilities. We focused on questions that would provide a wide insight into the common practices with regard to purchase, housing, use, health examination, monitoring and procedures at the end of experiments. Our hypothesis was that, compared to small laboratory animals, procedures related to FAs are less standardized and based more on facility-specific protocols, while the available recommendations of FELASA are not widely implemented. 

In Europe, the use, housing and welfare aspects of animals used for scientific purposes are regulated by Directive 2010/63/EU ([12]). The latter has been implemented into the national laws of all European Union member states.

In article 33 (1), the Directive states that all animals must be provided with an appropriate housing environment and necessary food, water and care. Additionally, in Annex III, the Directive specifies that all animal facilities must have a strategy to ensure the maintenance of an appropriate state of health that guarantees animal welfare and meets scientific requirements. The strategy must include regular health examinations as well as a microbiological surveillance program. Furthermore, for both small laboratory animals and farm animals, detailed recommendations are provided on the sampling of various species [16,23,24]. These are intended, on the one hand, to ensure the health status of the animals and, on the other hand, to enable the better assessment and comparability of scientific results. While these recommendations are not binding laws, they are widely used and applied in European small laboratory animal facilities. 

However, based on the long-lasting experience of the experts of the LaNiV network, and as indicated in the literature when our survey was prepared, it seems that the present recommendations for farm animals established by Rehbinder et al. between 1998 and 2000 [16,23] have not been widely applied thus far. A revision of the then-existing recommendations was only published after our survey was conducted in 2020 [13,14]. 

### 4.1. General Aspects

Most of our participants kept pigs, while the second most frequently kept animals were small ruminants, which is consistent with experimental animal statistics (BMEL, Daten zur Verwendung von Versuchstieren 2019, attachment 2, page 1). Unsurprisingly, a great number of our participants used FAs for translational medical research and education purposes, underlining the importance of these species as suitable translational models for human and veterinary medicine [3,4]. 

### 4.2. Purchase of Large Farm Animals

Following article 10 of EU Directive 2010/63, member states shall ensure that animals belonging to the species listed in Annex I may only be used in procedures where those animals have been bred for use in procedures (Directive 2010/63/EU of the European Parliament and of the Council of 22 September 2010 on the protection of animals used for scientific purposes). Contrary to small laboratory animals, such as mice and rats, neither pigs nor ruminants are listed in Annex I, as these species can be obtained from other sources. In accordance with the FELASA farm animal working group [13], our results confirm that FAs are predominantly purchased from livestock production. In pigs and small ruminants, most participants prefer to obtain the animals from one specific farm or breeder, which also confirms the results from the FELASA working group [14]. If purchased from a special breeder, pigs are mostly bought from a special breeder of experimental animals. In contrast, large ruminants are mainly bred within agricultural-based research facilities. Small ruminants are partly obtained from special [23] breeders and partly from in-house breeding. One reason for in-house breeding could be the limited availability of commercial animal breeders specialized in small and large ruminants, whereas miniature pigs can be obtained from commercial breeders specialized in experimental animals, even providing animals with an SPF (specific pathogen-free) status [25]. As an alternative to a commercial breeder, in-house breeding allows the effective control of age, sex, health status and preventive care to improve study design and scheduling [26]. 

If animals are purchased from livestock production breeders, breeding and producing facilities do not have to follow EU Directive 2010/63. Nevertheless, they must follow their local public legal requirements, which may differ between the European Union member states. Table S2 in the Supplementary Materials provides an overview of the legislation and recommendations for the breeding and housing of large farm animals in Germany. 

This overview highlights differences and potential limitations in legal regulations. In ruminants, for example, hygiene measures and management are not strictly regulated by law, but are mostly based on recommendations. Therefore, their health status could be unknown and hygiene management may vary from facility to facility, increasing the risk of introducing epizootic pathogens with the entry of new animals to the experimental facility. In contrast, small laboratory animals are bred under strict hygiene measures that ensure a SPF status of the animals. Therefore, small laboratory animals are usually delivered with an appropriate health certificate that includes their disease history over the previous six months, as recommended by [24]. 

The results of our survey show that a comparable number of participants ordered pigs either with or without a health certificate, whereas small ruminants were mostly purchased with a health certificate. Large ruminants are mostly purchased without a health certificate. For large ruminants used in agricultural research, specific pathogen-free hygiene status may be of secondary importance. Instead, they are housed in a conventional manner in which the absence of clinical signs of disease is sufficient. 

In general, most health certificates did not include detailed information on individual disease history or about serological, parasitological or bacteriological results, which is mandatory for rodents [24] and recommended for pigs, sheep and calves [16,23]. As recognized by the FELASA farm animal working group, existing FELASA recommendations for farm animals between 1998 [16] and 2000 [23] seemed unhelpful [13]. Therefore, they published updated recommendations for the health management of ruminants and pigs used for scientific purposes in 2020 [13]. These recommendations focus on general health management procedures, such as competent veterinary care, rather than testing an exhaustive list of pathogens in general [13]. If the new FELASA recommendations have more widespread application, further surveys may show improvements in farm animal health and welfare and the standardization of health management in research facilities that work with farm animals in Europe. 

If animals are delivered with an unknown health status and without an appropriate health certificate, clinical examination by a veterinarian and quarantine of the animals after entry to the research premises is highly recommended to protect the safety and health of the existing animal population in the facility. 

Following our survey, quarantine programs were used in approximately 50% of the participating facilities for all farm animal species. If animals were quarantined, this was mostly conducted in their own housing facility, with the exception of pigs, where quarantine is performed half in the research facility and half in the farm of origin. Regarding prophylactic treatments, the results were highly varied regarding choice, frequency and place in all farm animals kept under experimental conditions. This confirms the lack of widely used protocols for hygiene measures regarding the purchase and introduction of new animals to a facility [13]. In summary, their mostly unknown health status when animals enter these facilities and the lack of standardized quarantine procedures can cause health issues that are unrelated to the experiments that are performed, as already reported by Berset et al. [14]. Furthermore, this can cause hygiene hazards, with an impact on the results and reproducibility of experiments [13,16,23]. Therefore, the inspection of all animals procured from outside sources and testing for selected species-related diseases or conditions prior to purchase are recommended [13,26].

### 4.3. Husbandry of Large Farm Animals Kept for Experimental Reasons

To address this topic, we asked our participants to provide information on the hygiene measures applied within their housing facilities. The majority of our participants said that they changed their clothes and shoes before entering the facility, but a strict barrier was only reported by a few participants, mainly regarding pigs and small ruminants. For pigs and small ruminants, limited access was reported by most of participants, but this was not the case for large ruminants. Our results could be explained by the different research areas in which the animals are used. In contrast to large ruminants, small ruminants and pigs are often used in translational medical research and for educational purposes. Hygienic measures and restricted access might be highly important for these purposes. Large ruminants are primarily used for the categories of basic research and “protection of the natural environment in the interests of the health or welfare of human beings or animals” (BMEL, Daten zur Verwendung von Versuchstieren 2019, attachment 2, page 7). This was also confirmed by the results of our survey, as participants stated that they used large ruminants mainly for agricultural und for veterinary research. The animals here are presumably kept according to similar criteria as on farms and they are mostly conventional animals. These access restrictions on animal husbandry are at a different level. However, hygiene measures, such as the changing of shoes and clothes, seem to be standard if working with FAs. 

In our participating facilities, narrow internal health-monitoring programs are widely used for all farm animals. This implies that more emphasis is placed on hygiene issues during housing than on the health information of the purchased animals. However, the questions from our species-specific section, the majority of which were not answered, might indicate that hygiene monitoring either does not follow recommendations or standard lists, as already pointed out from the FELASA farm animal working group [13], or that participants did not answer the species-specific part due to other reasons—for example, because the size of the questionnaire exceeded their time capacities.

### 4.4. General Animal Safety Aspects If Using Large Female Farm Animals

Female sheep are mostly used for experimental purposes [14], as they are easier to handle and can be housed in groups, which also applies for pigs and large adult ruminants; however, the exclusion of a pregnancy might be important. 

Therefore, we asked our participants to provide information on the measures they implemented to exclude unwanted pregnancies in their experimental animals. Most participants received FAs without a declared pregnancy statement. Reported measures in our survey to exclude an unwanted pregnancy included ultrasound examination (pigs) or ultrasound and medicinal treatment (small ruminants). None of these methods was performed in large ruminants. This result reasonably reflects the different breeding systems. While in pigs and large ruminants artificial insemination is mostly performed, the existence of a pregnancy is most likely known at the time of purchasing the animals, and the risk of unknown pregnancies is limited. In contrast, natural mating is mostly performed in sheep, increasing the risk of unknown pregnancies. The unwanted use of pregnant animals in experiments must be avoided due to legal, ethical and animal protection aspects. Furthermore, pregnancy and birth can influence research results.

Therefore, the exclusion of pregnancy via blood analysis, ultrasound or medicinal treatment before including animals in a research project is highly recommended by the authors if female sheep are purchased from a conventional livestock farm. 

### 4.5. Approaches at the End of Experiments or after Experimental Usage of the Animals

With regard to the national implementation of the Directive on the protection of animals used for scientific purposes (63/22010 EU), we also asked our participants if they used any kind of tissue and organ sharing databases. In the sense of the principle of reduction and as all member states are engaged to establish programs for sharing the organs and tissues of animals that are killed, it is promising that a high number of our participants use programs to share organs and tissues. Interestingly, the most common way of sharing is the use of internal programs. This is potentially related to an easier distribution process and facility regulations (e.g., hygienic measures). 

In accordance with article 19 (2010/63/EU), setting animals free into a suitable habitat or rehoming are possible under specific conditions: Sufficient health status, no danger for public health or the environment and appropriate measures to safeguard animal wellbeing. These options at the end of a procedure are important, as animals may only be killed if there is a reasonable reason to do so. This includes the further necessary processing of organs or tissue in the context of an animal experiment. Based on the Directive, an animal should be killed at the end of procedures when it is likely to remain in moderate or severe pain, suffering, distress or lasting harm (art. 17, no. 2 2010/63/EU). Unlike small laboratory animals, the return to the food chain is of high interest as an option at the end of a procedure to kill FAs for a reasonable cause. In article 63 2010/63/EU (amendment of regulation (EC) no. 1069/2009), the legal possibility of returning farm animals to the food chain was provided. However, the transfer of farm animals after experimental use is only possible if it can be proven that the food derived from these animals is safe within the meaning of article 14 of regulation (EC) no. 178/2002. The responsibility to prove the safety must be taken by the research facilities or businesses involved in dispensing and receiving/accepting farm animals [27]. In the case of feeding trials with additives, which are not legally approved, article 3 (2) of regulation (EC) 1831/2003 must also be taken into account. 

In addressing whether farm animals in the participating institutes should be set free, rehomed or returned to the food chain, answers clearly showed that a lower number of our survey participants conducted private rehoming, particularly in large ruminants. This is probably related to the fact that farm animals are rarely kept as private pets. Our assessment of the subsequent commercial use of animals was determined by the number of responses, which confirmed the animals’ return to the food chain by in-house slaughtering or by giving FAs to third parties (slaughterhouses or fatteners). Interestingly, although participants returned the animals to the food chain after use by external slaughtering, only a low number did so with the presence of an official veterinary certification. In terms of protection claims, it is recommended that an official safety check is performed in agreement with the competent authority before animals are given to third parties for delivery back to the food chain [27]. 

### 4.6. Limitations

We must emphasize that this overview only provides results from participating institutes in Germany. There are no concrete numbers available in Germany on how many animal facilities with farm animals used for animal experiments exist. Since the survey was widely distributed and the experimental animal science institutions in Germany are very well networked, the results can, nevertheless, be considered significant, in our view. There were several further limitations to our study. As multiple answers were not possible in the SurveyMonkey platform, we had to provide the participants with as many answer combinations as possible, which made the survey very long and potentially tiresome to answer. This may explain the large number of incomplete surveys. In particular, our species-specific part regarding detailed in-house health monitoring, where all notifiable diseases in Germany and the FELASA mentioned diseases were listed, seemed to overwhelm the participants when filling out the survey. The lack of responses to this section may also be explained by the fact that this type of testing was not implemented in their hygiene-monitoring procedures. Finally, the number of participants using large ruminants was very low and may not be representative.

## 5. Conclusions

The aim of the survey carried out in this study was to identify the status quo and practical applied measures regarding the purchase and hygiene monitoring of FAs in order to improve animal welfare and scientific validity in Germany. 

The results of the survey show that, contrary to small laboratory animals, no standardized procedures were established regarding the purchase, housing and hygiene management of farm animals in the participating facilities in Germany. However, the facilities made purpose-bound decisions according to their own needs and individual work instructions. Strict pathogen lists, as in the case of small laboratory animals, do not seem to serve any purpose when using FAs. As FAs are important translational and comparative animal models, it is likely that their use as experimental animals will continue to increase in the near future. This underlines the need to provide support to FA users in developing practical hygiene programs that also have an impact on the farms of origin. This survey could help to critically screen individual facilities to identify hygiene risks, which can then be improved. The new FELASA recommendations [13] can hereby serve as guidance for the implementation of new hygiene management strategies. 

It is promising that practice measures related to reducing and refining, such as the sharing of organs and tissues, were already established in many facilities.

## Figures and Tables

**Table 1:** Age groups of animals kept experimentally. Distribution of housed aged groups for the different farm animals; “no information” means that a question was not answered.

Age Groups	Pigs	Small Ruminants	Large Ruminants
no information/no	15	11	5
young (before sexual maternity)	1	0	1
adult (after sexual maturity)	0	4	0
young and adult	12	8	3

**Table 2:** Housing capacities. Housing capacities for the different farm animals; “no information” means that a question was not answered.

Housing Capacities	Pigs	Small Ruminants	Large Ruminants
no information/no	15	11	5
<30	6	5	1
>30	3	5	1
>100	4	2	2

**Table 3:** Origin of pigs used for experimental research.

Special Breeder (*n* = 15)	*n*	Livestock Production (*n* = 23)	*n*
one specialized breeder for experimental animals	7	one livestock farm	14
own breeding	4	trader, obtaining pigs from one livestock farm	3
various breeders of experimental animals	2	various livestock farms	2
own breeding, various breeders for experimental animals or trader obtaining animals from various known breeders.	2	one livestock farm and own breeding	1
		one livestock farm and breeder of experimental animals	1
		various livestock farms and own breeding	1
		various, known livestock farms and trader, obtaining pigs from various known breeder of experimental animals	1

**Table 4:** Origin of small ruminants used for experimental research.

Special Breeder (*n* = 11)	*n*	Livestock Production (*n* = 18)	*n*
own breeding	5	one farm	11
own breeding and one breeder of experimental animals	2	various farms	2
one breeder of experimental animals	2	trader, obtaining pigs from various known farms	2
various breeders of experimental animals	2	own breeding and from various farms	1
		own breeding and from a trader obtaining small ruminants from one farm	1

## Data Availability

Not applicable.

## Supplementary Materials

Available online at https://www.mdpi.com/article/10.3390/ani11082158/s1. Table S1: Questions and answer possibilities from the survey, Table S2: Overview of the legislation and recommendations for the breeding and housing of large farm animals in Germany.
