# Supplementary material for: Large Farm Animals Used for Research Purposes: A Survey on Purchase, Housing and Hygiene Management
Source: Animals (Basel). 2021 Jul 21;11(8):2158. doi: 10.3390/ani11082158 (PMC8388472; doi:10.3390/ani11082158)
Supplement: Supplementary file 1 [file animals-11-02158-s001.zip › manuscript ID animals_1236343 Table S2, Supplementary Material.pdf]

**1. Purchase of animals used for experimental purposes**		
**Small laboratory animals**	Directive 2020/63 EU, Art 10: “1. Member States shall ensure that animals belonging to the species listed in Annex I may only be used in procedures where those animals have been bred for use in procedures”. Only applicable for mice, rats and others but not for pigs and large or small ruminants	
**Large farm animals**	FA can be obtained from livestock. General public laws and regulations for breeding units of livestock animals are applicable.	
**2. Legislation addressing hygiene issues for breeding and housing of small laboratory animals and farm animals in livestock or kept as experimental animals. Overview over German national (*) and applicable European legislation for experimental animals.**		
**Species**	**Applicable law/regulation/directive indicated by the authorities**	**Summary of required or recommended hygiene measures**
**Small laboratory animals**<br><br>**and**<br><br>**Large farm animals kept as experimental animals**	EU Directive EU 2010/63 [1]<br><br>Animal Protection Law* [2]<br><br>Tierschutzversuchstierverordnung* [3]	In article 33 (1): …all animals has to be provided with the appropriate housing, environment and necessary food, water and care….all animal husbandries must have a strategy to ensure the maintenance of a appropriate state of health that ensures animal welfare and meets the scientific requirements. The strategy has to include regular health examinations as well as a microbiological surveillance program.<br><br>more general information, no detailed hygiene monitoring aspects<br><br>more general information, no detailed hygiene monitoring aspects
**Farm animals in general**	Gesetz zur Vorbeugung vor und Bekämpfung von Tierseuchen (Tiergesundheitsgesetz - TierGesG)* [4]<br><br>Verordnung über anzeigepflichtige Tierseuchen (TierSeuchAnzV) [5]<br><br>Verordnung über meldepflichtige Tierkrankheiten [6]	regulate the prevention and measures of and against notifiable and reportable animal diseases<br><br>provides a list with notifiable animals disease in Germany<br><br>provides a list with reportable animals disease in Germany
**Small ruminants kept as farm animals**	No special legislation	
**Large ruminants kept as farm animals**	Tierschutz-Nutztierhaltungsverordnung (TierSchNutztV)* [7]	only applicable for calves, not for adult animals, general requirements for housing, space, no special hygiene requirements, control of calves , welfare twice a day
**Pigs kept as farm animals**	Tierschutz-Nutztierhaltungsverordnung (TierSchNutztV)* [7]	general requirements for housing, space, no special hygiene requirements, control of animals welfare once a day

	Regulation for hygiene issues of pig husbandries* [8]	internal controls to reduce animal disease intake risk,<br>control of animal in-and outcome,<br>veterinary heard care with clinical, examinations twice a year or once per mast round<br>Special examinations in suspected cases with focus on Schweinepest, African Schweinepest, Aujeszkysche disease and Brucellose
**1. Recommendations addressing hygiene issues for breeding and housing of small laboratory animals and farm animals in Germany before 2020**		
**Small laboratory animals**<br><br>**and**<br><br>**Farm animals kept as experimental animals**	EMPFEHLUNG DER KOMMISSION mit Leitlinien für die Unterbringung und Pflege von Tieren, die für Versuche und andere wissenschaftliche Zwecke verwendet werden [9]	general recommendations; quarantine, microbiological health monitoring, prophylaxis, daily health control and species specific general recommendations for farm animals, pigs and miniature pigs regarding vaccination, antiparasitic treatments
	„FELASA Recommendations for the health of mouse, rat,…colonies in breeding and experimental units" [10]	provide general recommendations and special pathogen list to be excluded for hygiene monitoring of experimentally housed mice and rats
	"FELASA Recommendations for the health monitoring of experimental units of calves, sheep and goats…" [11]	provide general recommendations and special pathogen list to be monitored as detailed recommendations for hygiene monitoring for calves, sheep and goats kept for experimental purpose
	"FELASA Recommendations for the health monitoring of breeding colonies and experimental units of cats, dogs and pigs…" [12]	provide general recommendations and special pathogen list to be monitored as detailed recommendations for hygiene monitoring for breeding and experimentally housing of cats, dogs and pigs
	Federation of European Laboratory Animal Science Associations recommendations of best practices for the health management of ruminants and pigs used for scientific and educational purposes [13]	published in 2021 with general and updated recommendations of health management for farm animals.<br>This was not applicable when we performed our survey.
**Small and large ruminants kept as farm animals**	Recommendations for hygiene measures for husbandry of ruminants 7.7.2014* [14]	focus on prevention and disease management of Q-fever and paratuberculosis: general recommendations and special remarks regarding vaccination

1. Directive 2010/63/EU of the European Parliament and of the Council. Available online: https://eur-lex.europa.
2. Tierschutzgesetz (TierSchG). **24.07.1972 last changes 19.06.2020**.
3. Verordnung zum Schutz von zu Versuchszwecken oder zu anderen wissenschaftlichen Zwecken verwendeten Tieren (Tierschutz-Versuchstierverordnung - TierSchVersV). **01.08.2013 last changes 31.08.2015**.
4. Gesetz zur Vorbeugung vor und Bekämpfung von Tierseuchen (Tiergesundheitsgesetz - TierGesG). **21.11.2018 last changes 20.11.2019**.
5. Verordnung über anzeigepflichtige Tierseuchen (TierSeuchAnzV). **23.05.1991 last changings 31.3.2020**.
6. Verordnung über meldepflichtige Tierkrankheiten. **11.02.2011**.
7. Tierschutz-Nutztierhaltungsverordnung (TierSchNutztV). **25.10.2001, last changes 29.01.2021**.
8. Verbraucherschutz, B.f.J.u. Schweinehaltungshygieneverordnung-SchHaltHygV. **07.06.1999; last changings 02.04.2014**.
9. EMPFEHLUNG DER KOMMISSIONvom 18. Juni 2007mit Leitlinien für die Unterbringung und Pflege von Tieren, die für Versuche und anderewissenschaftliche Zwecke verwendet werden. **18.07.2007**.
10. Mahler, M.; Berard, M.; Feinstein, R.; Gallagher, A.; Illgen-Wilcke, B.; Pritchett-Corning, K.; Raspa, M.; Revision, F.W.G. FELASA recommendations for the health monitoring of mouse, rat, hamster, guinea pig and rabbit colonies in breeding and experimental units. *Lab Anim-Uk* **2014**, *48*, 178-192, doi:10.1177/0023677213516312.
11. Rehbinder, C.; Alenius, S.; Bures, J.; de las Heras, M.; Greko, C.; Kroon, P.S.; Gutzwiller, A.; Hlth, F.W.G.A. FELASA recommendations for the health monitoring of experimental units of calves, sheep and goats - Report of the Federation of European Laboratory Animal Science Associations (FELASA) Working Group on Animal Health. *Lab Anim-Uk* **2000**, *34*, 329-350, doi:Doi 10.1258/002367700780387723.
12. Rehbinder, C.; Baneux, P.; Forbes, D.; van Herck, H.; Nicklas, W.; Rugaya, Z.; Winkler, G.; Hlth, F.W.G.A. FELASA recommendations for the health monitoring of breeding colonies and experimental units of cats, dogs and pigs - Report of the Federation of European Laboratory Animal Science Associations (FELASA) Working Group on Animal Health. *Lab Anim-Uk* **1998**, *32*, 1-17, doi:Doi 10.1258/002367798780559428.
13. Berset, C.M.; Caristo, M.E.; Ferrara, F.; Hardy, P.; Oropeza-Moe, M.; Waters, R.; Anim, F.W.G.F. Federation of European Laboratory Animal Science Associations recommendations of best practices for the health management of ruminants and pigs used for scientific and educational purposes. *Lab Anim-Uk* **2021**, *55*, 117-128, doi:Artn 00236772209444610.1177/0023677220944461.
14. Empfehlungen des Bundesministeriums für Ernährung und Landwirtschaft für hygienische Anforderungen an das Halten von Wiederkäuern. **07.07.2014**.
