# Supplementary material for: Large Farm Animals Used for Research Purposes: A Survey on Purchase, Housing and Hygiene Management
Source: Animals (Basel). 2021 Jul 21;11(8):2158. doi: 10.3390/ani11082158 (PMC8388472; doi:10.3390/ani11082158)
Supplement: Supplementary file 1 [file animals-11-02158-s001.zip › Supplements.pdf]

Table S1: Questions and answer possibilities from the survey

Part	Question	Possible answers
1	Please provide detailed information about the nature of your facility, the housed species, the housing capacities and the age groups of the housed animals.	
	Research focus	
	medical	no
		no information
		yes: translational medical research; veterinary medicine research; behaviour research; other topic
	agricultural	no
		no information
		yes: consumer protection; animal husbandry; animal nutrition; behavioural research; other topic
	breeding	no
		no information
		yes: own use; experimental animal breeder with delivery to other user
	housed age groups	no information
		young animals (before sexual maturity)
		adults (after sexual maturity)
		young and adults
	housing capacities	no information
		<30
		>30
		>100
2	Please indicate what measures are implemented for new animal arrivals at your facility(ies).	
	no access restriction	yes
		no
		no information
	strict barrier (clean/unclean side)	yes
		no
		no information
	restrictive personal access	yes
		no
		no information
	change of clothes and shoes	yes

		no
		no information
	hood/mouthguard/gloves	yes
		no
		no information
	showering	yes
		no
		no information
3	Please indicate the germ reduction procedures you have carried out.	
	ventilation	overpressure ventilation with HEPA filter
		overpressure ventilation without HEPA filter
		Vacuum ventilation with HEPA filter
		Vacuum ventilation without HEPA filter
		none
		no information
	water	Tap water
		Germ-reduced tap water
		no information
	ready feed	yes
		no
		no information
	roughage	yes
		no
		no information
	green fodder	yes
		no
		no information
	bedding	straw
		Germ-reduced straw
		none
		no information
4	Please provide information from where you purchase your farm animals.	
	special breeder	own breeding
		own breeding and breeder for laboratory animals,
		own breeding and varying laboratory animal breeders
		own breeding and trader obtaining from a constant

		breeder for laboratory animals,
		own breeding and trader obtaining from varying, known breeders for laboratory animals
		own breeding and trader obtaining from varying,unknown breeders for laboratory animals ,
		constant breeder for laboratory animals
		varying breeder for laboratory animals
		trader obtaining from constant breeder for laboratory animals
		trader obtaining from varying, known breeders for laboratory animals
		trader obtaining from varying, unknown breeders for laboratory animals
		no information
	livestock production	constant farm,
		varying farms
		trader obtaining from constant farm,
		trader obtaining from varying, known farms
		dealer obtaining from unknown varying farms,
		constant farm and own breeding
		varying farms and own breeding
		dealer obtaining from constant farm and own breeding
		dealer obtaining from varying, known farms and own breeding,
		trader obtaining from varying, unknown farms and own breeding
		constant farm and breeder for laboratory animals,

		constant farm and trader obtaining from a breeder for laboratory animals
		varying farms and varying breeders for laboratory animals,
		varying farms and trader obtaining from varying, known breeders for laboratory animals,
		trader obtaining from varying,unknown farms and varying, unknown breeders for laboratory animals
		no information
5	Please give information about the health status of the animals and performed hygiene monitoring.	
	transported separately when obtained from different origins	transport separately
		transport not separately
		no information.
	purchased with health certificate	yes
		no
		no information
	purchased without health certificate	yes
		no
		no information
	health certificate including the history of infectious diseases in the past	yes
		no
		no information
	health certificate including serological results	yes
		no
		no information
	health certificate including parasitological results	yes
		no
		no information
	health certificate including bacteriological results	yes
		no
		no information
	health certificate includes the exclusion of a pregnancy	yes
		no
		no information

	performance of ultrasound to exclude a pregnancy	yes
		no
		no information
	medicinal treatment to exclude a pregnancy	yes
		no
		no information
	separate housing of different genders to exclude a pregnancy	yes
		no
		no information
6	Please describe your quarantine and prophylactic measures and the restokking procedure of housing rooms	
	quarantine management	no quarantine
		Routine/Holding of origin
		Routine/in own holding (animal husbandry)
		in suspected cases in own animal husbandry
		no information
	prophylaxic treatments	none
		single antiparasitic treatment own animal husbandry
		repeated antiparasitic treatment own animal husbandry
		single antiparasitic treatment holding of origin
		repeated antiparasitic treatment holding of origin
		single vaccination in own animal husbandry
		repeated vaccination in own animal husbandry
		single vaccination in holding of origin
		repeated vaccination in holding of origin
		single vaccination and anitparasitic treatment in own animal husbandry
		repeated vaccination and anitparasitic treatment in own animal husbandry

		single vaccination and anitparasitic treatment in holding of origin
		repeated vaccination and anitparasitic treatment in holding of origin
		no information
	restokking procedure	yes
		no
		no information
7	Please describe the health and hygiene measures	
	general information	not established yet
		routinely
		in suspected cases
		no information
	animal section internally	never
		in suspected cases
		routinely
		in supected cases with histology
		routinely with histology
		no information
	animal section externally	never
		in suspected cases
		routinely
		in supected cases with histology
		routinely with histology
		no information
8	Please provide information about animal husbandry	
	single+stable (all year)	yes
		no
		no information
	group+stable (all year)	yes
		no
		no information
	group+stable (all year)+single possible	yes
		no
		no information
	group+stable+pasture (temporary)	yes
		no
		no information

	group+pasture (all year)	yes
		no
		no information
	Group+pasture (all year)+single housing possible	yes
		no
		no information
9	Please provide information about end of procedure or breeding exclusion (end of use)	
	program to share organs/tissues	no
		internal program
		external program
		internal and external programm
		no information
	animal release	no
		yes, private
		yes, commercial
		yes, private and commercial
		no information
	slaughter	no
		in-house slaughtering
		animal release and external slaughtering
		animal release and external slaughtering (with authority veterinarian certification)
		in-house slaughtering + external slaughtering
		in-house slaughtering + external slaughtering (with authority veterinarian certification)
		no information
